# Transmission route and reasons for HIV testing among recently diagnosed HIV patients in HIV-TR cohort, 2011–2012

**DOI:** 10.7448/IAS.17.4.19595

**Published:** 2014-11-02

**Authors:** Basak Dokuzoguz, Volkan Korten, Deniz Gökengin, Muzaffer Fincanci, Taner Yildirmak, Uzun Nuray Kes, Nuriye Tasdelen Fisgin, Dilara Inan, Haluk Eraksoy, Halis Akalin

**Affiliations:** 1 Infectious Diseases and Clinical Microbiology, Ankara Numune Education Training and Research Hospital, Ankara, Turkey; 2 Infectious Diseases, Marmara University Hospital, Istanbul, Turkey; 3 Infectious Diseases, Ege University Hospital, Izmir, Turkey; 4 Infectious Diseases, Istanbul Education and Research Hospital, Istanbul, Turkey; 5 Infectious Diseases, Okmeydaný Education and Research Hospital, Istanbul, Turkey; 6 Infectious Diseases, Þiþli Education and Research Hospital, Istanbul, Turkey; 7 Infectious Diseases, 19 Mayýs University Hospital, Samsun, Turkey; 8 Infectious Diseases, Akdeniz University Hospital, Antalya, Turkey; 9 Infectious Diseases, Istanbul Medical School, Istanbul University, Istanbul, Turkey; 10 Infectious Diseases, Uludað University Hospital, Bursa, Turkey

## Abstract

**Introduction:**

Routes of transmission and reasons for HIV testing are important epidemiologic data to analyze the epidemic and to tailor the response to AIDS. The aim of this study was to analyze reasons for testing and transmission ways of HIV among recently diagnosed HIV patients registered in the multicenter HIV-TR cohort in Turkey.

**Methods:**

Transmission ways and reasons for testing of all patients diagnosed in 2011 and 2012 were recorded on a web-based data collection system and were analyzed retrospectively.

**Results:**

The study included 693 patients (561 male, 132 female) from 24 sites. Reason for HIV testing was available in 640 patients (92%). The most common reason for HIV testing was diagnostic workout for other conditions or illness followed by patient-initiated testing. The reasons for testing were listed in Table 1. The most common routes of HIV transmission were heterosexual intercourse (62.7%) and sex among men who have sex with men (MSM) (22.6%). At the time of HIV diagnosis, the mean CD4 lymphocyte cell count was 355/mm^3^ (3–1433/mm^3^). Primary HIV infection was determined in 42/693 (6%) patients and 9/693 (% 1, 2) cases were considered “probable primary HIV infection.” The majority of the cases presented to a clinic for follow-up right after the diagnosis. On the other hand 32/616 (5.2%) patients delayed their presentation for more than 3 months. The longest delay was 11 months.

**Conclusions:**

The results of the database suggest that targeted testing is lacking in the country. The shift toward homosexual transmission during the last 2 years emphasizes the need for targeted interventions. Patients present relatively late and HIV infection could only be diagnosed when immunosuppression related findings appeared. Patient-initiated testing,an indicator of awareness, was very low suggesting a need to scale-up awareness raising interventions.

**Table 1:** Reasons for HIV testing

Reason for testing	N	%
Blood/organ donation	63	9.8
Prior to surgical/parenteral intervention	63	9.8
Premarital testing	20	3.1
Screening for pregnancy	11	1.7
Patient-initiated	98	15.3
Diagnostic workout for other medical condition/illness	312	48.8
Job application	8	1.3
Other	65	10.2
Total	640	100

